# The genome-wide relationships of the critically endangered Quadricorna sheep in the Mediterranean region

**DOI:** 10.1371/journal.pone.0291814

**Published:** 2023-10-18

**Authors:** Gabriele Senczuk, Marika Di Civita, Luigina Rillo, Alessandra Macciocchi, Mariaconsiglia Occidente, Giorgio Saralli, Valentina D’Onofrio, Tiziana Galli, Christian Persichilli, Claudio Di Giovannantonio, Fabio Pilla, Donato Matassino

**Affiliations:** 1 Department of Agriculture Environment and Food Science, University of Molise, Campobasso, Italy; 2 Consortium for Experimentation, Dissemination, and Application of Innovative Biotechniques, (ConSDABI), Benevento, Italy; 3 Agenzia Regionale per lo Sviluppo e l’Innovazione dell’Agricoltura del Lazio (ARSIAL), Roma, Italy; 4 Istituto Zooprofilattico Sperimentale del Lazio e della Toscana M. Aleandri (IZSLT), Roma, Italy; Sul Ross State University, UNITED STATES

## Abstract

Livestock European diffusion followed different human migration waves from the Fertile Crescent. In sheep, at least two diffusion waves have shaped the current breeds’ biodiversity generating a complex genetic pattern composed by either primitive or fine-wool selected breeds. Nowadays most of the sheep European breeds derive from the second wave which is supposed to have largely replaced oldest genetic signatures, with the exception of several primitive breeds confined on the very edge of Northern Europe. Despite this, some populations also in the Mediterranean region are characterised by the presence of phenotypic traits considered ancestral such as the policeraty, large horns in the ram, short tail, and a moulting fleece. Italy is home of a large number of local breeds, albeit some are already extinct, others are listed as critically endangered, and among these there is the Quadricorna breed which is a four-horned sheep characterised by several traits considered as ancestral. In this context we genotyped 47 individuals belonging to the Quadricorna sheep breed, a relict and endangered breed, from Central and Southern Italy. In doing so we used the Illumina OvineSNP50K array in order to explore its genetic diversity and to compare it with other 41 breeds from the Mediterranean region and Middle-East, with the specific aim to reconstruct its origin. After retaining 32,862 SNPs following data filtering, the overall genomic architecture has been explored by using genetic diversity indices, Principal Component Analysis (PCA) and admixture analysis, while the genetic relationships and migration events have been inferred using a neighbor-joining tree based on Reynolds’ distances and by the maximum likelihood tree as implemented in treemix. The Quadricorna breed exhibit genetic diversity indices comparable with those of most of the other analysed breeds, however, the two populations showed opposing patterns of genetic diversity suggesting different levels of genomic inbreeding and drift (*F*_*IS*_ and *F*_*ROH*_). In general, all the performed genome-wide analyses returned complementary results, indicating a westward longitudinal cline compatible with human migrations from the Middle-East and several additional genetic footprints which might mirror more recent historical events. Interestingly, among the Italian breeds, the original Quadricorna (QUAD_SA) first separated showing its own ancestral component. In addition, the admixture analysis does not suggest any signal of recent gene exchange with other Italian local breeds, highlighting a rather ancestral purity of this population. On the other hand, both the neighbor-joining tree and the treemix analysis seem to suggest a proximity of the Quadricorna populations to breeds of South-Eastern Mediterranean origin. Although our results do not support a robust link between the genetics of the first wave and the presence of primitive traits, the observed genetic uniqueness together with the inferred phylogeograpic reconstruction would suggest an ancient presence of the Quadricorna breed in the Italian Peninsula. Because of this singularity, urgent conservation actions are needed in order to keep the breed and all related cultural products alive.

## Introduction

In livestock, SNP genotyping arrays are among the most widely used genetic tools for a wide range of studies including understanding species’ evolutionary mechanisms and domestication processes, discovering signature of selection, disentangling the nature of complex traits through genome-wide association studies as well as improving selection methods for animal production in the so-called genomic selection [1, 2]. Such widespread use is mostly related to SNP arrays efficiency in terms of cost and time, but it is also due to their repeatability, which enables the construction of huge genotypic datasets. However, this latter point is not always trivial due to many factors that include different SNPs densities, continuous updates in the reference assembly and strand flipping issues might arise. Recently, because of a recognized strand flipping error, we retracted a work just few days after its publication in this journal [3, 4]. Here we corrected by updating the variant allele codes of the whole dataset and re-analysed the genome-wide breeds diversity such as follow.

Since Neolithic revolution, domestic animals followed human migration routes and spread throughout the world. For many livestock species, this processes have been shown to have occurred in distinct historical periods by following different colonisation routes leading to a vast array of breed diversity well adapted to specific local environments [5–7], that is to say with a high "capacity for constructivism", understood as the tendency of organisms to actively participate in the construction of a specific "bioterritory" rather than simply "adapting", establishing a vital relationship with it to achieve maximum fitness [8–11].

Compared to other domestic species such as cattle, goat and dogs, sheep (*Ovis aries*) have been shown to have retained much higher genetic diversity conforming with a wider heterogeneous pre-domestication populations, combined with less severe post-domestication bottlenecks [12, 13]. Moreover, after sheep domestication, at least two principal waves from the Fertile Crescent prompted the diffusion of an early hair-type (~10.500 YBP) followed by a later wool-type diffusion into Europe [14, 15]. An additional migration wave involving fat-tailed and fat-rumped sheep probably occurred about 3.500 YBP determining their expansion across North Africa, Middle-east and Asia [16–18].

In Europe, most of the genome of the first migration wave (hair type sheep), has been later replaced, generating a well-defined geographic cline from Middle-East to Balkan and continental Europe [19]. However, several relict primitive populations are still reared in scattered areas of Northern Europe [14] while different insular mouflon populations have also retained ancestral genetic diversity. These peripherical breeds apart from sharing a similar genotype and retrotype, are also recognized as primitive by possessing several morphological traits considered ancestral such as large horns in the ram, a short tail, and a moulting fleece [20]. In particular, the common presence of four-horned phenotypes has been argued to be present in short-tailed prehistoric sheep in Northwest-Europe (Iceland and British Isles) and in primitive breeds of Southern Europe (Cyprus and Spain), while the four-horned Jacob sheep is believed to be possibly linked to Middle-East [14, 21–23].

Concerning the Italian peninsula, a high level of genetic diversity with a strong phylogeographic structuring has been observed [24]. All genetic studies to date, identified a complex background compatible with a late migration triggered by wool production [25, 26]. For example, the presence of fine wool sheep was already documented in the Greek and Roman times and it is believed that starting from this period, they spread throughout Europe and crossed with other native breeds giving rise to the Spanish Merino breeds [16, 24]. Nowadays, Italy is home to more than 60 breeds, many of them reduced to small local populations and listed as critically endangered [27]. Instead, autochthonous breeds and their relative products, represent an important resource not only for their cultural and economic heritage but also as a source of genetic diversity important to face the new millennium challenges of livestock. So far, local breeds are experiencing an intense crisis due to their replacement with exotic and more productive breeds and as a consequence many native livestock populations have already become extinct [28]. In particular, it is estimated that as regards the Italian sheep populations, as many as 48% fall into the ’damaged’ risk category and 30% into the ’critical’ one [29]. In such a context, there is a growing urgency to perform genomic assessments of local breeds in order to explore their level of genetic diversity and eventually to improve adequate conservation and breeding strategies. Moreover, local breeds being well adapted to marginal areas characterised by poor quality feed and low input conditions, they potentially function as a reservoir of genes able to confer high “capacity of constructivism", important to counteract all related deleterious effects caused by climate and environmental changes. Indeed, many recent studies focusing on local breeds have highlighted a number of potential genes involved in disease resistance, tolerance to harsh conditions and adaptation to low input and sustainable systems [30].

Among the most critically endangered Italian local breed is the Quadricorna sheep which is constituted by a very residual population reared in Central-Southern Italy, counting in 2022 only 2 farms, one located in Colliano (SA, Campania) and the other in Monte San Giovanni Campano (FR, Lazio) with 33 (20 females, 13 males) and 23 (21 females, 2 males) heads respectively. The sheep population called “Pecora Quadricorna” has been registered since 2006 in the Regional Voluntary Register of Lazio established and from 2018 to the National Registry, but currently, the Herd Book is still lacking. The most characteristic trait of the breed is the polyceraty, hence the name Quadricorna which means four-horned. Worldwide, there are numerous sheep populations showing polycerism [31] being present in both Northern European populations (sheep of Ireland, Felsen and Soa, Faröe, Shetland, Orkney and Man islands) and in populations of Tartaria, Nepal, Himalaya, Caucasus, Tunisia, Algeria, and in the Americas (Navajo-Churro). In Italy, four-horned sheep was mainly common among the Pagliarola breed in Abruzzo and in those of the Emilian Apennines [31]. Although still debated, several hypotheses have been argued to explain the origin of four-horned sheep. According to Marchi [31], the presence of multiple horns was common in all sheep derived from the paleo-Egyptian sheep and referable to *Ovis longipes*. On the other hand, other authors, primarily Sanson [32], argued that the origin of polycerate sheep can be traced back to the breed of Syria (*Ovis aries asiatica*), as described by Lemoigne [33].

To disentangle such a complexity and to shed light on the origin of the critically endangered Quadricorna sheep, we genotyped the two remaining populations from Central and Southern Italy using the Illumina OvineSNP50K BeadChip. Therefore, the main aims of this study are (i) to explore the genomic architecture of the Quadricorna population, (ii) to compare it with other Mediterranean breeds in order to gather insights into a more complete evolutionary picture and to (iii) assess whether the presence of considered primitive related traits is also accompanied by ancestral genetic diversity.

## Materials and methods

### Ethics statement

Blood samples were collected from sheep during normal health prophylaxis procedures by the Istituto Zooprofilattico Sperimentale del Lazio e della Toscana, in agreement with the recommendations of European Union Directive 2010/63/EU, to ensure appropriate animal care.

### Sampling and genetic diversity indices

A total of 47 blood samples belonging to the Quadricorna sheep breed (31 females, 12 males and 4 individuals with no sex information) were stored and selected for the genotyping. These samples included 22 individuals belonging to Colliano (QUAD_SA) and 25 to Monte San Giovanni Campano (QUAD_FR). The two examined farms pursue similar breeding conditions in a semi-wild state with small additions of flaked feed when fresh forage is scarce.

The Illumina OvineSNP50K BeadChip was used to genotype the Quadricorna sheep populations while the raw genotyped data for 41 breeds (1,119 individuals) were retrieved from previous works [12, 15, 24, 34–40] (S1 Table).

Using the software plink v1.9 [41], all genotypes were filtered for missing call rate (—geno 0.1), minor allele frequency (—maf 0.05) and missing genotype rate (—mind 0.1, removing 1 sample), obtaining a final dataset consisting of 32,862 SNPs and 1,165 samples.

For each breed expected (H_e_) and observed (H_o_) heterozygosity and the inbreeding coefficient (F_*IS*_) was calculated using the R package dartR [42]. In addition, inbreeding coefficient based on runs of homozygosity (F_ROH_) was calculated for each breed using the following parameters: the minimum length of the ROH was set to 4000 (—homozyg-kb), the SNPs density was set to 100 (—homozyg-density) with a maximum gap between consecutive homozygous SNPs of 1,000 (—homozyg-gap), one missing SNP (—homozyg-window-missing), up to one possible heterozygous genotype was allowed in the ROH (—homozyg-window-het) and the minimum number of SNPs within a ROH was set to 50 (—homozyg-window-snp). Thus, the final inbreeding coefficients (F_ROH_), have been calculated using the autosomal length as implemented in the *genome_wide* function of the R package *detectRUNS* [43].

Finally, the contemporary effective population size (*N*_*e*_) for each breed was calculated using the software GONe [44] which uses the linkage disequilibrium (LD) between markers to infer very recent demographic changes. All parameters except the recombination rate (hc = 0.01) were set by default.

### Genetic structure and breed relationships

To visualise the genetic differentiation among breeds, a principal component analysis (PCA) was performed using plink. The PCA was performed through a supervised approach using the “—pca-cluster” flag in plink v1.9, in order to account for breed heterogeneity and outliers. Results of the centroids were then plotted using the R package *ggplot2* [45].

The maximum clustering likelihood approach as implemented in the software admixture [46], was used to estimate the genetic structure, testing K values ranging from 1 to 50. For each K, we run three independent iterations, setting the cross-validation error procedure to 10-fold in order to choose the most informative number of K repartitions. The number of K before the separation of the two Quadricorna populations, was also used to plot the mean of the ancestral components in a pie fashion into a geographic map.

The phylogenetic relationships among breeds were inferred using the “dist.genpop” function implemented in the R package adegenet [47]. Starting from a genpop object, this function generates the genetic Reynolds’ distance matrix from which we derived the neighbor-joining tree using the "ape" package [48].

To assess the genetic drift and migration events among breeds, the maximum likelihood dendrogram was inferred using the software treemix [49]. The analysis was performed taking into account LD over blocks of 500 contiguous SNPs, using the Soay breed as outgroup, while allowing 10 migration events over 5 iterations. To evaluate the best number of migration edges, the R package OptM [50] was used to fit the parametric models to the likelihood values across runs.

## Results

**Fig 1:**
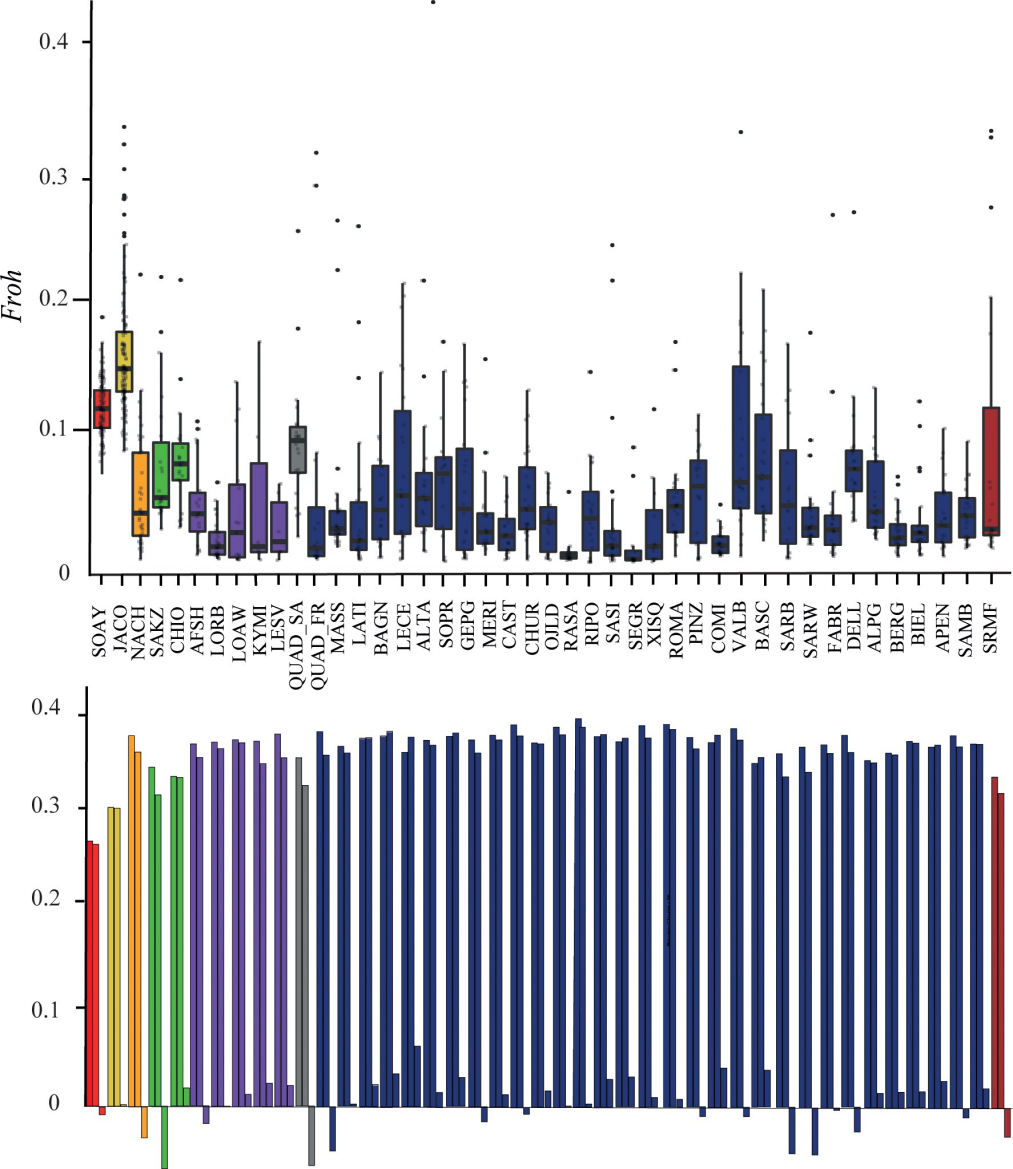
Inbreeding coefficients based on *Froh* (a) and *Fis* (b). Colours of the breeds are depicted according to the main genomic repartition at K = 9.

All genetic diversity indices are reported in Table 1 and Fig 1. The minimum value of H_e_ and H_o_ was observed in the Soay breed, while the highest values were observed in Rasa Aragonesa. Concerning the F_IS_, the highest value was observed in the Leccese breed while the most negative value was for Sakiz. In general, we observed a higher amount of negative F_IS_ values in ether breeds considered primitive (SOAY, AFSH, SAKZ and SRMF) or with known recent history of admixture (BASC, SARB, SARW and NACH). Interestingly both Quadricorna populations showed negative Fis values (Fig 1B). The inbreeding estimated using runs of homozygosity (F_ROH_) showed the highest values in Jacob and Soay, while the minimum value was observed in the Rasa Aragonesa (RASA) breed. The two Quadricorna populations showed different values of inbreeding, lower in the QUAD_FR than in the QUAD_SA (Fig 1A).

**Table 1:** Genetic diversity indices for all the analysed breeds. Observed (Ho) and expected heterozygosity and their relative standard deviation (SD), inbreeding coefficients (*Fis* and *Froh*) and effective population size (*Ne*).

	Breed	Code	Location	N	Ho	SD	He	SD	*Fis*	*Froh*	*Ne (hc = 0*.*01)*
**1**	Navajo-Churro	NACH	USA	36	0.380	0.160	0.363	0.138	-0.032	0.053	36.983
**2**	Jacobs	JACO	USA	124	0.306	0.172	0.305	0.168	0.002	0.162	187.148
**3**	Soay	SOAY	Scotland	110	0.277	0.186	0.273	0.182	-0.008	0.122	90.263
**4**	Ojalada	OJLD	Spain	24	0.389	0.148	0.381	0.122	0.001	0.030	360.448
**5**	Castellana	CAST	Spain	22	0.392	0.154	0.380	0.124	-0.007	0.025	1,255.78
**6**	Churra	CHUR	Spain	28	0.372	0.150	0.372	0.130	0.015	0.050	59.858
**7**	Segureña	SEGR	Spain	12	0.390	0.175	0.378	0.127	0.010	0.017	360.066
**8**	Sasi Ardi	SASI	Spain	24	0.373	0.150	0.377	0.126	0.030	0.038	1,401.47
**9**	Rasa Aragonesa	RASA	Spain	20	0.397	0.149	0.389	0.115	0.003	0.009	604.682
**10**	Xisqueta	XISQ	Spain	23	0.392	0.146	0.386	0.117	0.009	0.029	214.643
**11**	Ripollesa	RIPO	Spain	22	0.379	0.149	0.381	0.122	0.028	0.041	120.389
**12**	Roja Mallorquina	ROMA	Spain	24	0.378	0.161	0.366	0.135	-0.010	0.046	143.814
**13**	Sardinian Mouflon	SRMF	Sardinia	24	0.339	0.184	0.322	0.160	-0.029	0.087	25.589
**14**	Sardinian Ancestral Black	SARB	Sardinia	20	0.369	0.181	0.343	0.148	-0.048	0.056	64.844
**15**	Sardinian White	SARW	Sardinia	24	0.371	0.161	0.363	0.137	-0.002	0.036	127.547
**16**	Valle del Belice	VALB	Sicily	24	0.351	0.159	0.357	0.140	0.037	0.094	146.58
**17**	Pinzirita	PINZ	Sicily	24	0.373	0.147	0.381	0.122	0.040	0.054	764.388
**18**	Barbaresca	BASC	Sicily	24	0.362	0.184	0.338	0.155	-0.048	0.076	19.151
**19**	Comisana	COMI	Sicily	24	0.387	0.153	0.376	0.126	-0.009	0.012	300.952
**20**	Sambucana	SAMB	Piedmont	24	0.373	0.159	0.372	0.131	0.020	0.037	70.971
**21**	Delle Langhe	DELL	Piedmont	24	0.355	0.169	0.353	0.145	0.015	0.076	35.121
**22**	Biellese	BIEL	Piedmont	22	0.370	0.157	0.371	0.130	0.028	0.030	845.332
**23**	Bergamasca	BERG	Lombardy	24	0.375	0.155	0.373	0.129	0.016	0.021	662.625
**24**	Alpagota	ALPG	Veneto	24	0.363	0.166	0.361	0.139	0.016	0.047	135.776
**25**	Massese	MASS	Tuscany	24	0.368	0.163	0.361	0.139	0.002	0.048	123.658
**26**	Fabrianese	FABR	Marche	23	0.380	0.166	0.363	0.137	-0.025	0.039	38.604
**27**	Appenninica	APEN	Umbria	24	0.382	0.158	0.370	0.131	-0.009	0.034	134.192
**28**	Sopravissana	SOPR	Marche	24	0.378	0.146	0.382	0.122	0.030	0.066	401.338
**29**	Merinizzata	MERI	Abruzzo	20	0.380	0.157	0.375	0.127	0.013	0.034	652.246
**30**	Gentile Puglia	GEPG	Apulia	24	0.375	0.164	0.362	0.138	-0.015	0.057	77.305
**31**	Altamurana	ALTA	Apulia	22	0.375	0.158	0.370	0.133	0.009	0.060	107.107
**32**	Leccese	LECE	Apulia	23	0.362	0.147	0.378	0.125	0.062	0.076	79.333
**33**	Bagnolese	BAGN	Campania	23	0.379	0.145	0.384	0.119	0.034	0.046	301.001
**34**	Quadricorna	QUAD_SA	Campania	22	0.356	0.194	0.328	0.163	-0.061	0.092	86.718
**35**	Laticauda	LATI	Campania	24	0.377	0.151	0.377	0.125	0.022	0.045	84.885
**36**	Quadricorna	QUAD_FR	Latium	25	0.384	0.169	0.360	0.140	-0.045	0.055	106.657
**37**	Kymi	KYMI	Greece	6	0.374	0.223	0.351	0.150	0.024	0.050	96.016
**38**	Lesvos	LESV	Greece	6	0.381	0.221	0.356	0.146	0.021	0.026	321.966
**39**	Chios	CHIO	Greece	23	0.337	0.172	0.336	0.152	0.018	0.077	187.915
**40**	Sakiz	SAKZ	Turkey	22	0.347	0.200	0.319	0.169	-0.064	0.076	39.390
**41**	Local Awassi	LOAW	Israel	24	0.375	0.153	0.372	0.128	0.013	0.040	194.998
**42**	Afshari	AFSH	Iran	25	0.370	0.165	0.356	0.141	-0.018	0.045	49.458
**43**	Lori Bakhtiari	LORB	Iran	30	0.372	0.154	0.366	0.134	-0.001	0.017	116.938

The contemporary effective population size (N_e_) is reported in Table 1. The minimum value was observed in BASC while the maximum value in SASI. The two Quadricorna populations showed moderate Ne estimates with the QUAD_FR displaying a higher value (106) than the QUAD_SA (86).

**Fig 2:**
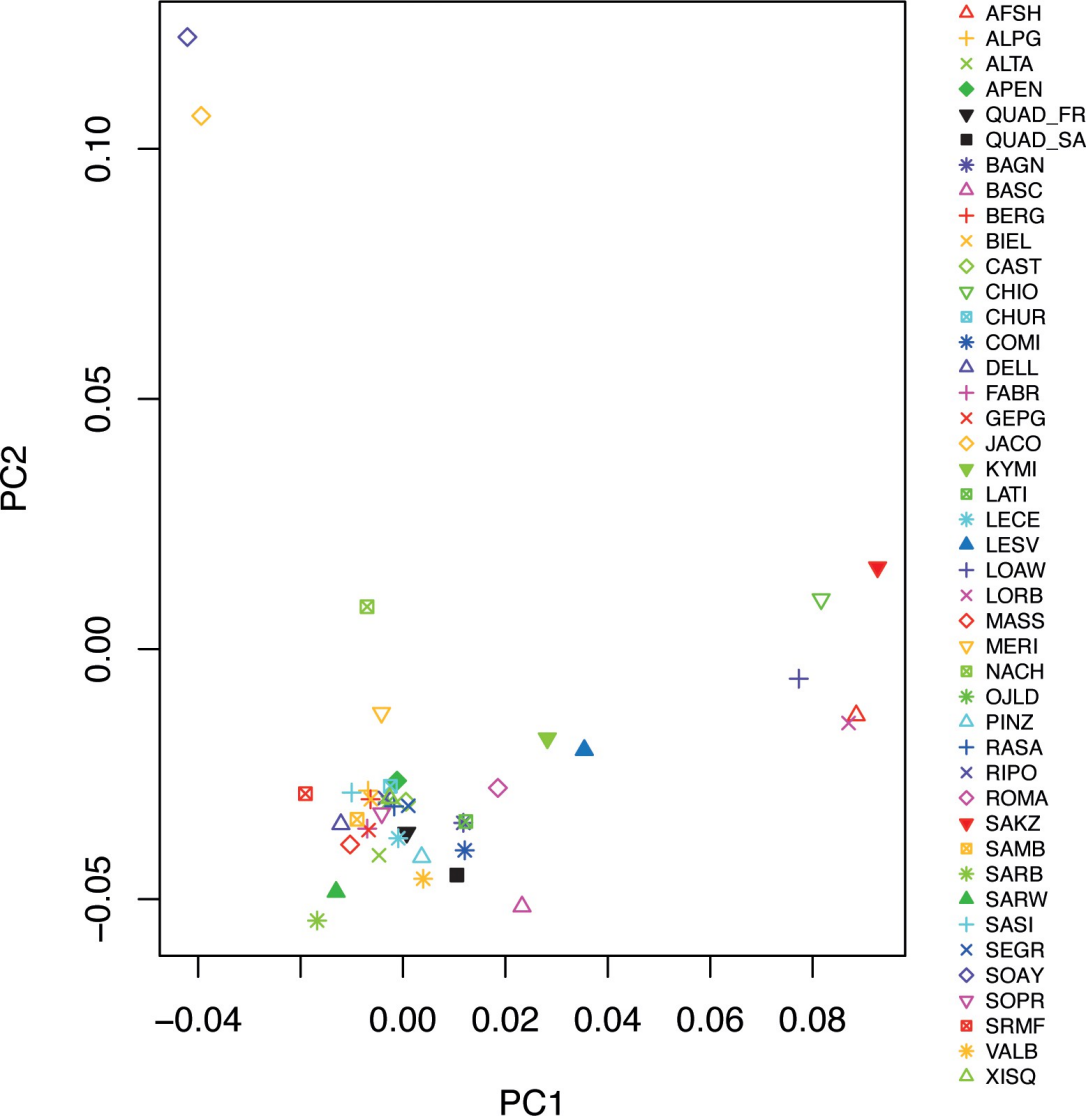
Supervised Principal Component Analysis (PCA) of all the analysed sheep breeds grouped according to their geographic origin.

The supervised PCA is reported in Fig 2. The first principal component which accounted for 12.18% of the total variance, distinguishes all breeds from Middle-East and Eastern Mediterranean areas (AFSH, LOAW, KYMI, SAKZ and LORB) from all the other breeds. This component also highlights a genetic gradient along the East-West axis from Balkan to Italian Peninsulas. The two Quadricorna populations display a quite different pattern being QUAD_SA closer to eastern breeds while QUAD_FR to southern Italian breeds. On the other hand, the second principal component which accounted for 9.39% of the total variance, separated the two primitive breeds of Soay and Jacob.

**Fig 3:**
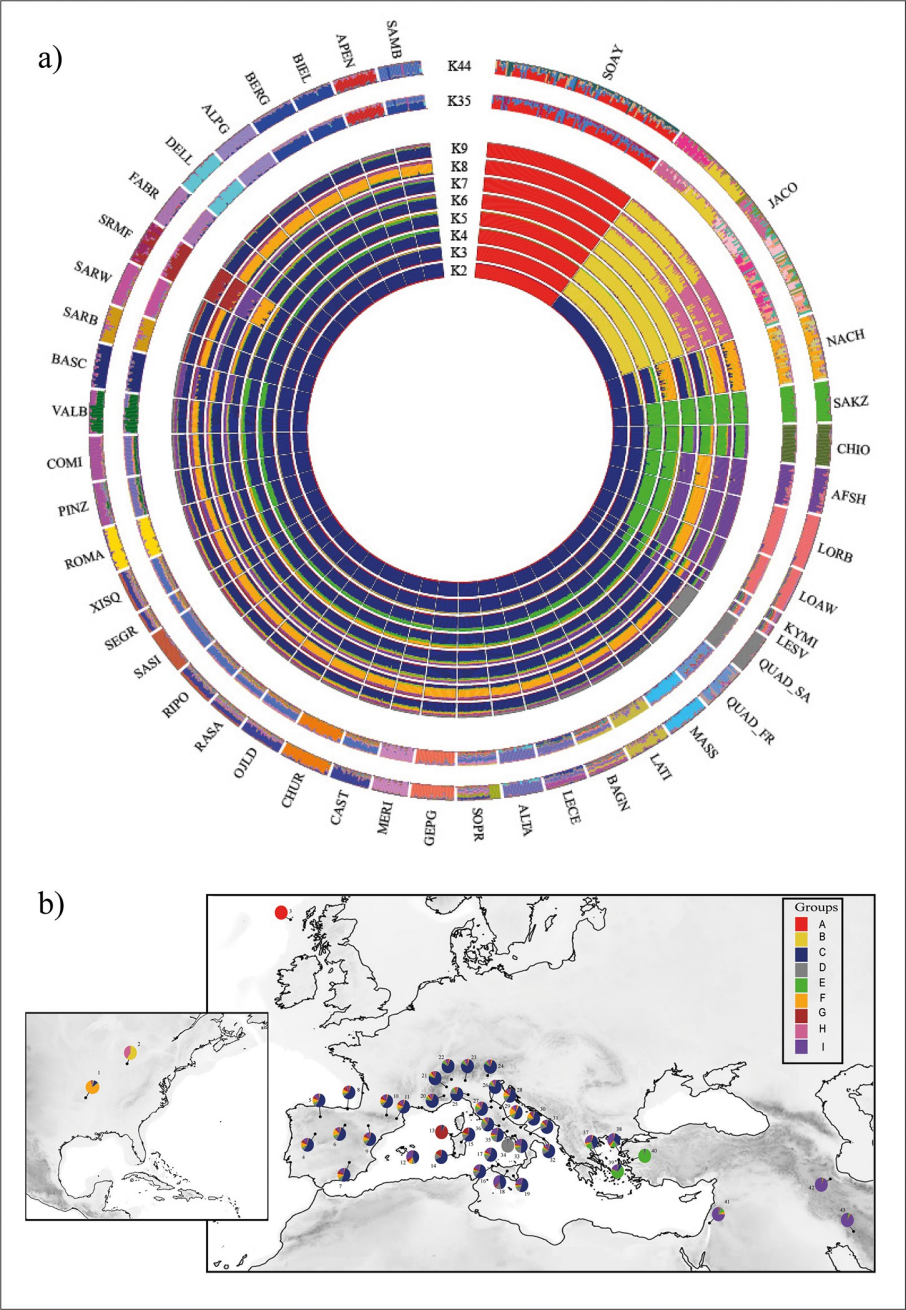
Results of the admixture analysis for *K* values ranging from 2 to 9, 35 and 44 (a). For K = 9, the genomic components are shown on a geographic map (b). See Table 1 for a full definition of the breeds.

The genetic repartition as highlighted by the admixture analysis showed a best K value at 44 (S1 Fig) where almost all breeds showed their own genetic pattern while for other breeds a marked sub-structuring is observable (Fig 3A). The admixture model, assuming two ancestral populations (K = 2), first separated the Soay from all the other breeds, while at K = 3, the Jacob breed is splitted in a different cluster. The two Quadricorna populations separated with all the other breeds at K = 9, however, at this K value, they display a different genetic admixture pattern (Fig 3B) with the QUAD_SA population showing a rather homogeneous pattern while the QUAD_FR showed more mixed components up to K = 40.

**Fig 4:**
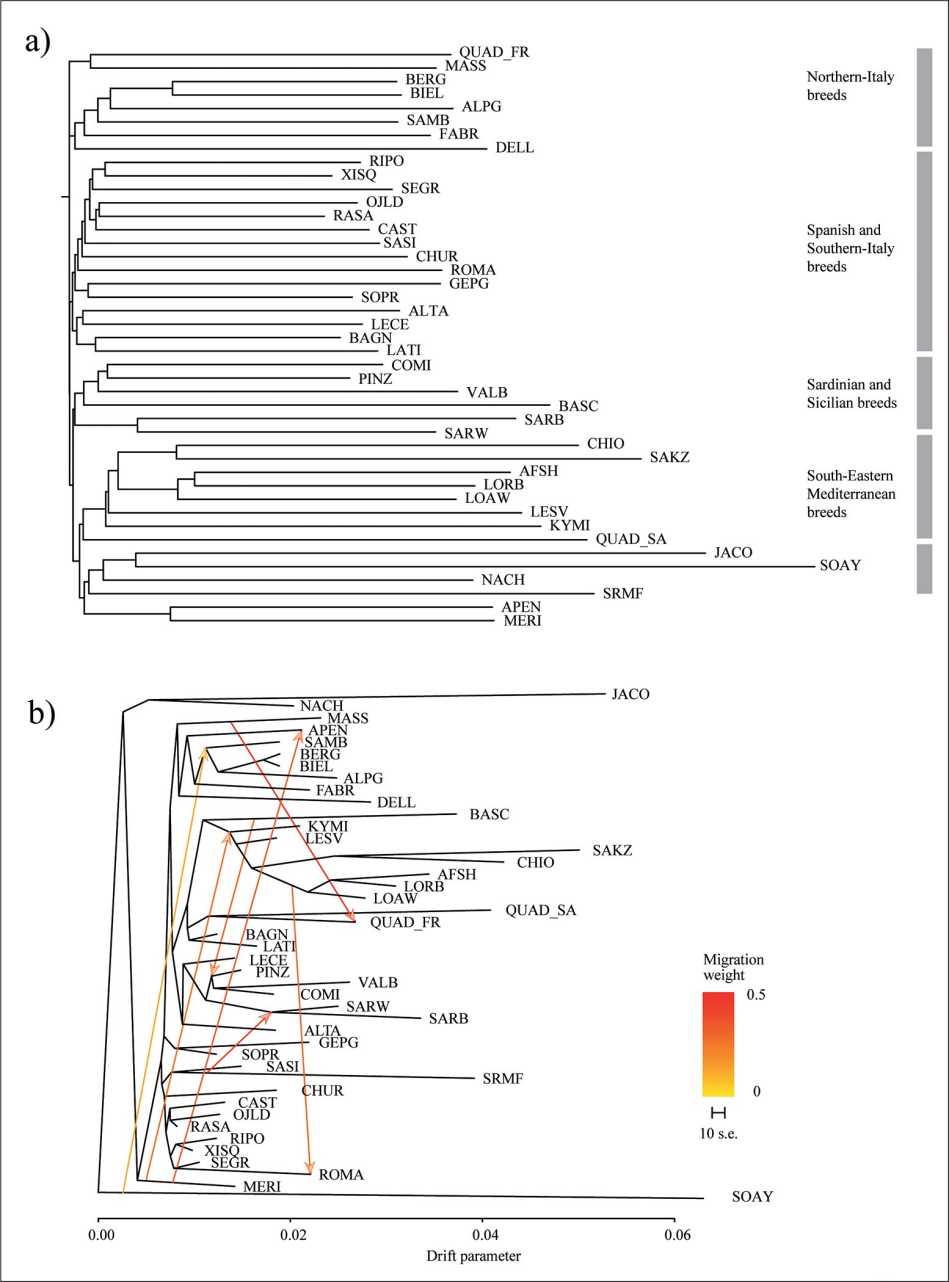
Genetic relationships among breeds as inferred by the neighbor-joining tree based on Reynold’ distances (a) and by the maximum likelihood treemix analysis (b). The best number of migration events is shown with relative arrows. Colours of the breeds are depicted according to the main genomic repartition at K = 9. For a full definition of the breeds see Table 1.

The neighbor-joining tree based on Reynolds’ distances, basically corroborates all the aforementioned results as for example confirming the basal genetic relationship between the Soay and Jacob breeds, but at the same time highlights some genetic relationships not clearly detected in the previous analyses (PCA and admixture). Indeed, the QUAD_FR is closely clustered with the Massese breed while the QUAD_SA is placed in a basal position of a group which includes Greek and Middle-East breeds (Fig 4A). Additional groups according to both breed history and geographic origin can be detected. Italian insular breeds from Sicily and Sardinia (SARW, SARB, BASC, VALB, PINZ and COMI) cluster together. Another group which includes Southern Italian breeds (LATI, BAGN, LECE and ALTA) is positioned in a basal position of a larger cluster which includes the Italian Merino-derived (SOPR and GEPG) and all the Spanish breeds. Finally, a group including all breeds from Northern Italy is also distinguished.

Finally, the treemix analysis showed several genetic relationships and migration edges useful to have a more resolute picture of the general historical evolutionary processes. Indeed, it is well known that dichotomous bifurcations do not always correctly describe the genetic relationships among populations at the intraspecific level [49]. In our case, although most of the geographically recognized groups highlighted by previous analyses remained almost unchanged, the inclusion of 7 migrations according to the likelihood fitted using the *optm* function, allowed us to obtain a more complete interpretation of split and genetic exchange events. For example, according to genetic drift, the two Quadricorna populations clustered together while a migration edge from MASS to QUAD_FR was highlighted. Concerning the topology, also in this analysis, the Quadricorna breed is grouped in a genetic cluster including all South-Eastern Mediterranean breeds, including BAGN and LATI. The strongest migration signals were found from SRMF to SARW and SARB, from MERI to APEN, from BASC, that in this analysis clusters with South-Eastern Mediterranean breeds, and the Sicilian breeds (COMI, PINZ and VALB) and from Middle-East (AFSH, LORB and LOAW) to ROMA. A weaker migration edge is also assigned from SOAY to Northern Italian breeds (BERG, BIELL and SAMB).

## Discussion

In a previous work we recently published in this journal, proudly and even a little surprised, we described the alleged ancestry of an endangered native breed (Quadricorna). Multiple convergent historical and morphological evidence moved towards an obvious and charming story to tell. However, only a few days after publication, we recognized a strand flipping error that strongly compromised the results by returning a substantial overestimation of the overall differentiation. Yet, despite the major oversight we also realised how we stumbled over the so-called confirmation bias. Behind this, we have re-analysed the whole dataset and although the divergences have largely diminished, we still found interesting patterns of ancestry which would seem to indicate a particular uniqueness of the Quadricorna breed with genomic relation with South East Mediterranean breeds.

### Genome-wide diversity and breed relationships

The genetic indices for the two Quadricorna populations in terms of expected and observed heterozygosity resulted in line with values of other local breeds in the Italian Peninsula (Table 1). Also, the effective population size and inbreeding coefficients were comparable with those observed in most of the analysed breeds. However, the two Quadricorna populations displayed slightly different genetic indices with the QUAD_FR showing higher diversity indices (H_o_ and H_e_), effective population size (*N*_*e*_) and lower inbreeding coefficients (*F*_*IS*_ and *F*_*ROH*_). Different patterns of genetic diversity indices between populations of the same breed are not surprising and have been commonly reported in many livestock species including local [30] and cosmopolitan breeds [51, 52]. Especially when considering residual populations at extinction risk and composed by few herds, the genetic drift can quickly act by reducing allele frequencies and increasing homozygosity. In addition, historical documentations have reported the past outcrosses with the Massese breed occurred for the QUAD_FR population, on the other hand, the QUAD_SA it remained isolated, maintaining a purer condition and with reduced genetic variability. In this context, the genetic assessment is of primary importance in order to establish conservation and recovery plans that take into account the maintenance of adequate genetic diversity.

On the whole, the genomic architecture of the analysed breeds resolutely reflects a genetic cline from East to West (Fig 3B) compatible with human migrations from the Middle-East, occurred after domestication [14, 15]. However, additional genetic signatures might mirror more recent historical events, generating alternative patterns of genetic diversity. As an example, within the first ten K values, a rather marked homogeneity can be found in both Spanish and Central and Southern Italian breeds. This result is not surprising since the Central and Southern Italian Peninsula has had centuries-old Spanish domination, releasing a genetic imprint fairly common especially in modern Italian Merino-derived breeds [35].

The population genetics analyses and phylogeographic reconstructions returned complementary pictures concerning the relationships of the two Quadricorna populations from Central and Southern Italy with all the other breeds. According to the PCA, three principal groups can be recognized based on breed geographic origin (Fig 2). Although the two Quadricorna breeds cluster within other European breeds, the QUAD_SA population resulted closer to South-Eastern Mediterranean breeds. This pattern is also in accordance with the neighbor-joining tree constructed using Reynolds’ distances and the ADMIXTURE analysis. Both analyses showed a genetic relatedness of the Quadricorna breed with both Greek and Anatolian populations (CHIO, KIMI, LESV, LOAW, and SAKZ), and the Middle-East breeds (AFSH and LORB). These results are particularly in line with several authors [32, 33], which argued that the origin of polycerate sheep can be traced back to the breed of Syria (*Ovis aries asiatica*). It is also interesting to note that in the admixture analysis, the QUAD_SA population is the first among the Italian breeds in showing its own ancestral component (K = 9, Fig 3). In addition, while QUAD_FR showed an admixed pattern according to recent outcrosses, QUAD_SA does not display any signal of recent admixture with other Italian breeds reared in neighbouring areas, pointing out a rather ancestral purity of this population. The genetic isolation of these residual Quadricorna populations might have been reinforced by the traditional vertical transhumance, that in turn, is based on the proximity between winter and summer pastures. Such a marginal pastoral system might have prevented large genetic exchanges as occurred for Merino-derived breeds in which the horizontal transhumance involving movements covering long distances, have favoured strong genetic connections among herds in the so called ’’Merinization’’ processes [53].

The observed genetic uniqueness together with the inferred phylogeograpic reconstruction pointed to a proximity of the Quadricorna populations to breeds of South-Eastern Mediterranean origin. To address such outcomes, a possible hypothesis can be related to a diffusion from the Middle-East probably as a consequence of the increased marine trade from the Hellenistic to the Roman periods. For example, written sources about livestock trade are reported in the *De re Rustica* of Lucius Junius Moderatus Columella (AD 4 – c.70), documenting the presence in the Italian Peninsula of livestock varieties from the Middle-East [54 and references therein]. Additional evidence coming from zooarchaeological and ancient DNA, showed similar outcomes, not only concerning the introduction of fine-fleeced sheep [55, 56].

From a morphological point of view, the Quadricorna shares several primitive traits (policeraty and hair-type) with those breeds that can be traced back to the first wave. Indeed, four-horned sheep were historically widely distributed in Europe, due to advantages in the size and arrangement of the horns in anti-predatory strategies [29]. Subsequently, this trait has become rare in both ewes and rams due to the long-term artificial selection [20, 21, 57]. To conclude, the results we reported do not seem to establish a firm link between the genetics of the first wave and the presence of primitive traits. On the other hand, the genomic relation of the Quadricorna breed with breeds of the South-Eastern Mediterranean region would suggest an ancient presence of this breed in the Italian Peninsula. Future studies would be desirable in order to shed light into the mode and the time of diffusion of this ancient breed, moreover we believe that our results should be considered in light to plan adequate conservation measures in order to recover the population and to valorise their related cultural and commercial products.

## Supporting information

S1 Fig Cross-validation error for the admixture analysis for K values ranging from 2 to 50. (TIF)

S1 Table Names, codes, geographic origin and all references of the analysed breeds. (DOCX)
