# Supplementary figures and images for: The genome-wide relationships of the critically endangered Quadricorna sheep in the Mediterranean region
Source: PLoS One. 2023 Oct 18;18(10):e0291814. doi: 10.1371/journal.pone.0291814 (PMC10584175; doi:10.1371/journal.pone.0291814)

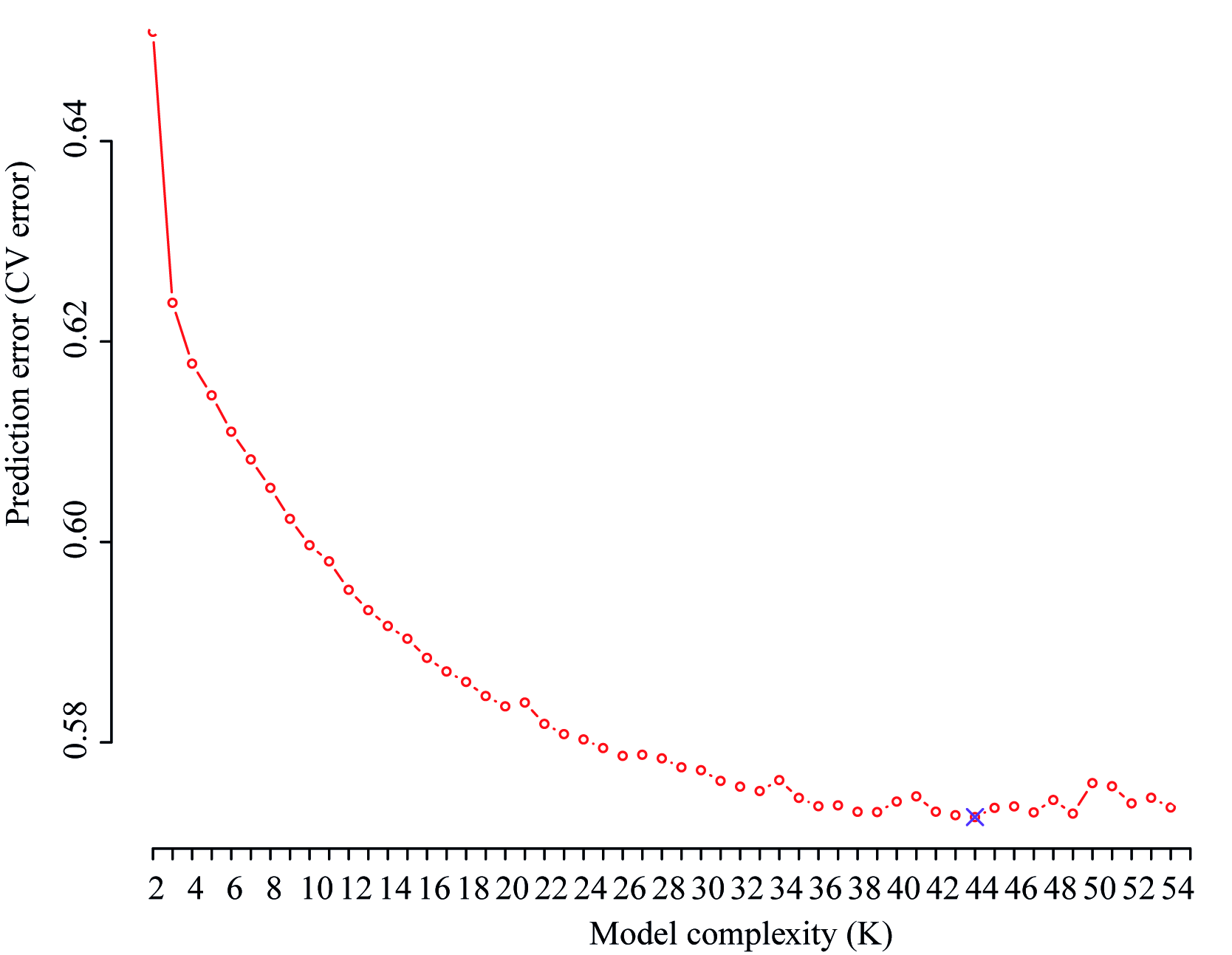

Supplement: S1 Fig — (TIF) [file pone.0291814.s001.tif]
