# Supplementary material for: The genome-wide relationships of the critically endangered Quadricorna sheep in the Mediterranean region
Source: PLoS One. 2023 Oct 18;18(10):e0291814. doi: 10.1371/journal.pone.0291814 (PMC10584175; doi:10.1371/journal.pone.0291814)
Supplement: S1 Table — (DOCX) [file pone.0291814.s002.docx]

**S1 Table.** Names, codes, geographic origin and all references of the analyzed breeds.

| **Breed/population** | **Breed code** | **Origin** | **References** |
| --- | --- | --- | --- |
| Afshari | AFSH | Iran | Kijas et al.,2012 |
| Alpagota | ALPG | Italy N | Ciani et al., 2013 |
| Altamurana | ALTA | Italy S | Kijas et al., 2012 |
| Appenninica | APEN | Italy C | Ciani et al., 2013 |
| Bagnolese | BAGN | Italy S | Ciani et al., 2013 |
| Barbaresca | BASC | Italy-Sicily | Mastrangelo et al., 2017 |
| Bergamasca | BERG | Italy N | Ciani et al., 2013 |
| Biellese | BIEL | Italy N | Ciani et al., 2013 |
| Castellana | CAST | Spain | Kijas et al., 2012 |
| Chios | CHIO | Greece | Kijas et al., 2012 |
| Churra | CHUR | Spain | Kijas et al., 2012 |
| Comisana | COMI | Italy-Sicily | Kijas et al., 2012 |
| DelleLanghe | DELL | Italy N | Ciani et al., 2013 |
| Fabrianese | FABR | Italy C | Ciani et al., 2013 |
| Gentile Puglia | GEPG | Italy S | Ciani et al., 2013 |
| Jacobs | JACO | U.S. | Kijas et al., 2016 |
| Kymi | KYMI | Greece | Ciani et al., 2020 |
| Laticauda | LATI | Italy S | Ciani et al., 2013 |
| Leccese | LECE | Italy S | Kijas et al., 2012 |
| Lesvos | LESV | Greece | Ciani et al., 2020 |
| Local Awassi | LOAW | Israel | Seroussi et al., 2017 |
| Lori Bakhtiari | LORB | Iran | Moradi et al., 2012 |
| Massese | MASS | Italy C | Ciani et al., 2013 |
| Merinizzata | MERI | Italy C | Ciani et al., 2015 |
| Navajo-churro | NACH | U.S. | Kijas et al., 2016 |
| Ojalada | OJLD | Spain | Kijas et al., 2012 |
| Pinzirita | PINZ | Italy-Sicily | Ciani et al., 2013 |
| Quadricorna (Frosinone) | QUAD_FR | Italy C | This study |
| Quadricorna (Salerno) | QUAD_SA | Italy S | This study |
| Rasa Aragonesa | RASA | Spain | Kijas et al., 2012 |
| Ripollesa | RIPO | Spain | Manunza et al., 2016 |
| Roja Mallorquina | ROMA | Spain | Manunza et al., 2016 |
| Sakiz | SAKZ | Turkey | Kijas et al., 2012 |
| Sambucana | SAMB | Italy N | Ciani et al., 2013 |
| Sardinian Ancestral Black | SARB | Italy-Sardinia | Kijas et al., 2012 |
| Sardinian Mouflon | SRMF | Italy-Sardinia | Ciani et al., 2015 |
| Sardinian White | SARW | Italy-Sardinia | Ciani et al., 2013 |
| Sasi Ardi | SASI | Spain | Ruiz‑Larrañaga et al., 2018 |
| Segureña | SEGR | Spain | Manunza et al., 2016 |
| Soay | Soay | Scotland | Kijas et al., 2012 |
| Sopravissana | SOPR | Italy C | Ciani et al., 2013 |
| ValledelBelice | VALB | Italy-Sicily | Ciani et al., 2013 |
| Xisqueta | XISQ | Spain | Manunza et al., 2016 |
|  |  |  |  |
